# The Effect of Sleeve Pattern and Fit on E-Textile Electromyography (EMG) Electrode Performance in Smart Clothing Design

**DOI:** 10.3390/s21165621

**Published:** 2021-08-20

**Authors:** Gozde Goncu-Berk, Bilge Guvenc Tuna

**Affiliations:** 1 Department of Design, UC Davis, 1 Shields Ave, Davis, CA 95616, USA; 2 Department of Biophysics, Medical Faculty, Yeditepe University, 26 Ağustos Yerleşimi, İnönü Mah. Kayışdağı Cad., Ataşehir, Istanbul 34755, Turkey; bilge.tuna@yeditepe.edu.tr

**Keywords:** e-textiles, electromyography, CAD embroidery, clothing fit

## Abstract

When e-textile EMG electrodes are integrated into clothing, the fit of the clothing on the body, and therefore its pattern and cut become important factors affecting the EMG signal quality in relation to the seamless contact between the skin and the e-textile electrode. The research so far on these effects was conducted on commercially available clothing or in tubular sleeve forms for arms. There is no study that investigated different clothing pattern and fit conditions and their effect on e-textile EMG electrode performance. This study investigates the effect of clothing pattern and fit in EMG applications using e-textile electrodes integrated onto the sleeves of custom drafted t-shirts in set-in and raglan sleeve pattern variations. E-textile electrode resistance, signal-to-noise ratio (SNRdB), power spectral density and electrode–skin impedance are measured and evaluated in set-in sleeve and raglan sleeve conditions with participants during a standardized arm movement protocol in comparison to the conventional hydrogel Ag/AgCl electrodes. The raglan sleeve pattern, widely used in athletic wear to provide extra ease for the movement of the shoulder joint, showed superior performance and therefore indicated the pattern and cut of a garment could have significant effect on EMG signal quality in designing smart clothing.

## 1. Introduction

Electromyography (EMG) is a diagnostic recording system routinely used in clinical applications to measure electrical activity of striated muscles as voltage and get information about the health condition of neuron groups that stimulate these muscles. Surface EMG (sEMG), which can be applied wirelessly, is widely used to study the dynamic muscle activity where the signals from muscles are registered noninvasively from the skin surface by electrodes to evaluate muscle activity without interfering with movements. In sEMG applications, electronic textile (e-textile) electrodes present multiple advantages including the possibility of being integrated into clothing for continuous muscle activity tracking when compared with the conventional, single use, hydrogel Ag/AgCl electrodes that require skin preparation. E-textile electrodes also offer the advantages of softness, flexibility, air permeability, multi-use and self-administration without medical assistance. In a comprehensive review by Guo et al. [1] embroidery [2,3]; knitting and weaving [2]; coating, screen printing [4] and bonding conductive materials are reported as the main methods of fabricating e-textile sEMG electrodes. Research also showed that the embroidery method provided repeatable and highly accurate results in manufacturing e-textile sEMG electrodes [5,6].

The major drawback in embedding e-textile electrodes into clothing is the loss of contact between skin and the electrode surface due to movements of the body and clothing in dynamic conditions where clothing may not behave and change in shape corresponding exactly to the body movement. Martin et al. discussed the effect of body size and motion in sensor and electrode placement on clothing [7].

Studies exploring e-textile sEMG electrodes embedded in different clothing forms report on the importance of the interaction between the electrode and body during movement. According to Finni et al. good fit of shorts provided good contact between e-textile electrodes and the skin, and prevented electrode displacement during joint movement [8]. However, authors did not provide a clear discussion of what constitutes as ‘good fit’. Another study by Taelman et al. similarly noted the fit of shirts as critical to reduce misalignment artifacts such as the muscle to electrode and electrode to electrode distances [9]. Authors suggested the fixed position of the e-textile sEMG electrode relative to the monitored targeted muscle in the design of the shirts and quantified the displacement of the shirt relative to the skin. Markers were placed on certain anatomical landmarks on the skin and on corresponding locations on the shirt to measure the displacement during shoulder movement and the influence of the amount of displacement on the EMG signal. The authors concluded that when the displacement of the shirt relative to the muscle is within 2.5 cm, the EMG signals were acceptable while the displacements of electrodes on the bone were not acceptable. In addition, e-textile sEMG electrode size and locations have been investigated. Kim, Lee and Jeong found out that signal-to-noise ratio of EMG signals increased significantly as the e-textile electrode, fabricated from silver and carbon conductive sheet, diameter increased [10]. On the other hand, as the e-textile diameter increased, the trade-off was the increase of the cross talk between the e-textile electrodes.

Other studies attempted to improve e-textile electrode and skin contact through different mechanisms. The first strategy was to add thickness between the electrode and the base fabric through embossed embroidery patterns or filling inserts such as foams, which may end up with uneven surfaces and altered the silhouette in tight-fitting clothing [10]. Liu, Zhu and Xia attempted to enhance the contact between e-textile electrocardiography (ECG) electrodes and the skin by covering the woven electrode adhered on the fabric surface by an 8-mm-thick sponge and by optimizing the shirt fit to minimize the textile displacements at the region of interest during moderate body movements [11].

Comert et al. compared knitted e-textile electrode paddings of different thicknesses and softness in retrieving ECG signals and reported that regardless of the clothing pressure exerted on electrodes, using paddings considerably improves signal quality and lowers motion artefacts [12]. Cho et al. employed e-textile ECG electrodes embroidered into an inflated shape to improve skin contact and non-elastic base fabric under the e-textile electrode to reduce signal-to-noise from stretch of the clothing during wearer’s movements [13]. Authors integrated these e-textile electrodes into different conditions created by elastic straps embedded in a shirt in horizontal, cross and x forms and compared the dynamic displacement of electrodes relative to the movement of the upper limbs and the torso in these conditions. The study concluded the shirt condition with cross form elastic strap performed the best in reducing the influences of motion artefacts and skin resistance.

The second strategy to improve e-textile electrode and skin contact was adjusting clothing tightness or, in other words, clothing fit, which is directly related to clothing pattern and cut [4]. Kim, Lee and Jeong investigated the effect of pressure between the skin and e-textile electrode by reducing the pattern in the width direction of leg sleeves from 0% to 30% to create a snugger fit and therefore increased clothing pressure applied over the electrodes. The authors recommended that clothing pressure over an electrode of more than 10 mm Hg for a textile-based electrode with a diameter of 20 mm was comparable to a traditional Ag/AgCl electrode [10]. Similarly, An et al. studied the effect of clothing pressure on e-textile ECG electrodes and concluded with an optimal pressure of 30 mmHg after which the participants felt uncomfortable [4]. Comert et al. also compared the effect of pressure applied on e-textile ECG electrodes in the arm sleeve form [12].

When e-textile EMG electrodes are integrated into clothing, the fit of the clothing on the body becomes an important factor directly affecting the EMG signal quality [13]. Fit of the clothing and therefore pattern and cut directly affect the contact area and the pressure between the skin and e-textile electrode, as well as the signal-to-noise ratio and electrical resistivity of e-textile EMG electrodes. However, the research so far focused on these effects through commercially available clothing or in tubular sleeve forms for arms. There is no study that investigates different clothing pattern and fit conditions. This study investigates the effect clothing pattern and cut in EMG applications using e-textile electrodes integrated onto the sleeves of custom drafted t-shirts in set-in and raglan sleeve pattern variations. E-textile electrode resistance, signal-to-noise ratio (SNRdB), power spectral density and electrode–skin impedance are measured and evaluated in set-in sleeves and raglan sleeves with participants during a standardized arm movement protocol in comparison to the conventional hydrogel Ag/AgCl electrodes.

## 2. Materials and Methods

### 2.1. Materials

The custom-fitted t-shirts with different sleeve variations were prototyped using a one-way stretch, polyester single-jersey knit fabric. For sportswear, knitted fabrics are widely preferred based on their unique characteristics such as elasticity, wrinkle resistance and ease of care [14], superior comfort qualities of water vapor permeability, air permeability, thermal conductivity and moisture management [15]. Synthetic fibers are considered as better alternatives compared to other fibers in active sportswear due to their heat and moisture management capabilities. Polyester is especially a popular synthetic fiber with its high dimensional stability, high strength and resistance to mold, organic solvents and alkalis [16].

In manufacturing the e-textile EMG electrodes, 100% polyamide core, silver-plated, 2-ply Madeira HC 12 (<100 Ω/m) conductive embroidery thread was used. This particular thread was preferred since it is specifically developed for embroidery applications transforming Statex’ silver plated Shieldex thread. A prior study also proved superior compatibility of Statex Shieldex thread in embroidery applications of e-textiles [17].

### 2.2. Material Characterization

Physical and mechanical properties of the knit fabric were measured. The fabric thickness was measured according to the ASTM D1777-96 (2015) standard; mass per unit are (weight) of the fabric was measured according to the ASTM D3776 standard; linear density of the fabric was determined. Bursting strength properties and elasticity of the knit fabric were determined using a Titan Universal Test device with 50 N load cell, consequently according to TS 393 EN ISO13938-1 and EN 14704-2005 standards.

The mechanical characteristics of the conductive thread such as thickness (dtex) and twist direction were determined. Electrical characterization of the conductive thread was performed in three repeats and then statistically analyzed. Electrical resistance of the thread was determined with the four-point probe method, while the signal-to-noise ratio (SNRdB) was measured using a function generator and oscilloscope.

### 2.3. Prototype Development

**Figure 1:**
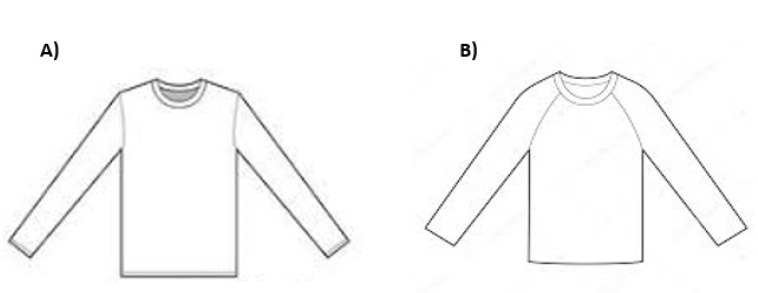
(**A**) T-shirt with set-in sleeve style, (**B**) t-shirt with raglan sleeve style.

The t-shirts were prototyped in two different sleeve pattern variations of set-in and raglan sleeve styles (Figure 1). Set-in sleeve and raglan sleeve patterns were selected as they are widely used in athletic wear and as they create different fit conditions around the shoulder and arm.

**Figure 2:**
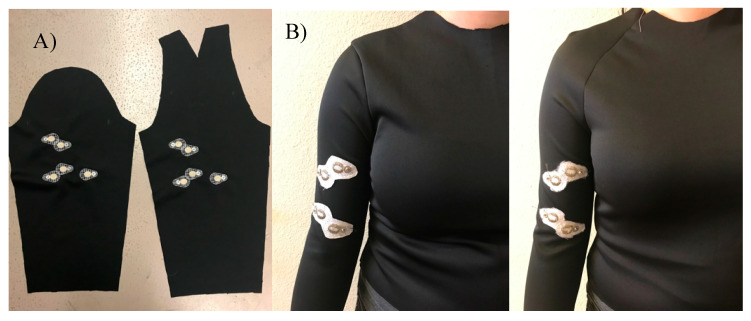
(**A**) Set-in and Raglan sleeves with embroidered electrodes, (**B**) The t-shirt prototypes constructed with set-in and raglan sleeve styles.

Two t-shirt patterns with set-in sleeves and raglan sleeves were custom drafted for one male and two female participants whose body measurements were retrieved using a Size Stream 3D body scanner (Figure 2). Waist, bust, neck, armhole, bicep and wrist circumferences, shoulder width and length, bust to bust length, arm length, half-back center, side neck to bust length and mid-shoulder to waist measurements were used to draft the t-shirt patterns for each participant. T-shirt patterns with set-in sleeves and raglan sleeves were then manually drafted based on the extension percentage of the knit fabric in course direction (138.3%) (BS EN 14704-2005 standard). A total of six custom-fitted t-shirt prototypes (one with set-in sleeve, one with raglan sleeve) were produced for the 3 participants.

**Figure 3:**
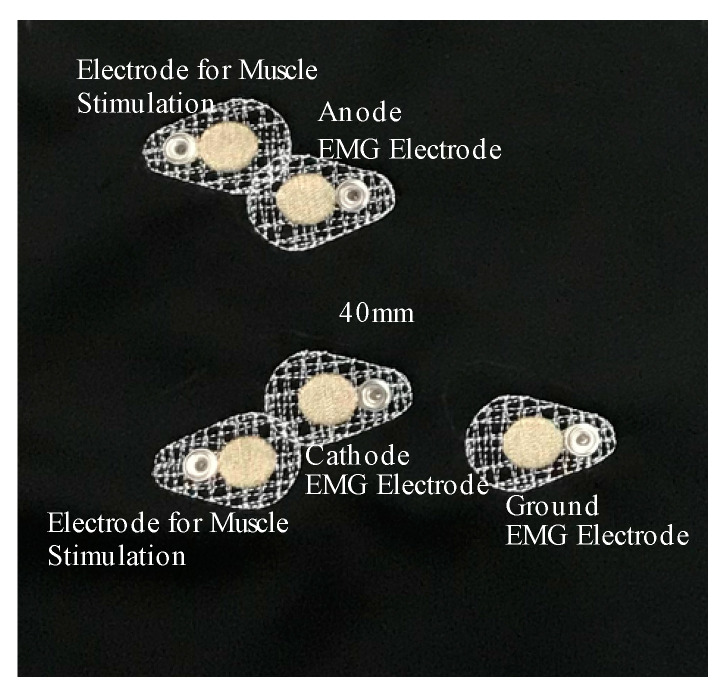
Placement of embroidered electrodes relative to each other.

E-textile EMG electrodes were embroidered on the right sleeve pattern of each t-shirt prototype using the polyamide silver-plated Madeira HC 12 (<100 Ω/m) thread and a ZSK Sprint CAD embroidery machine. EMG electrodes were digitally modelled using Tajima DG15 by Pulse embroidery software with the satin stitch type which is a series of flat stitches that completely cover a section of the fabric. During the embroidery process under-stitching with non-conductive thread was used to create a stabilized fabric surface, then the electrode surface was embroidered on top with the Madeira HC12 conductive thread. The under-stitching also helped to add extra raised surface for the electrodes which was critical for better electrode–skin contact. Two embroidered EMG electrodes (anode and cathode) with a 20-mm diameter were located 40 mm apart from each other on the sleeve pattern to correspond to the location of the Biceps Brachii muscle of the arm; one embroidered EMG electrode (ground) was located to correspond to the Humerus elbow bone; two additional electrodes were embroidered for use to stimulate muscles (Figure 3). The electrodes were embroidered on the inner surface of the sleeve for direct contact with the skin and male side of metal snaps were clipped on the embroidered electrodes on the outer surface of the sleeve for connection to the Delsys snap EMG sensor system.

### 2.4. Experimental Protocol

Experiments were conducted based on the procedure approved by Istanbul Technical University IRB with a total of five participants. Body Mass Indexes (BMIs) of all participants, demographic information, major body measurements and clothing sizes are listed in the below Table 1. Clothing sizes of the participants were determined based on visual evaluation of the garment fit by an expert scholar in clothing design due to limitation of the ASTM D5585-21 D6240/D6240M-12(2021) e1 standards [18] in accommodating variety in body measurements of individual participants. To determine clothing size, participants were fitted to a store bought long-sleeve top from a worldwide known sportswear company and the fit was evaluated based on smoothness of the fit with comfort and without any restrictions in movements, position of the structural seam lines and the balance of the garment on the body without fabric strains or wrinkles [19,20,21].

Out of these five participants, one male and two female participants were body-scanned and their body measurements were used to custom draft the two t-shirt patterns with set-in and raglan sleeves. In addition to the three body-scanned participants, one female and one male participant with matching sizes to the initial three body-scanned participants were recruited to analyze the conditions where the t-shirt was slightly snug and slightly loose within the same size limits compared to the custom-fit conditions. In Table 1, major body measurements that affect sleeve and shoulder fit are included to provide a quantitative comparison of custom fit and non-custom fit participants.

EMG measurements were taken from the participants under three conditions: 1. Bare skin with conventional hydrogel Ag Ag/Cl electrode; 2. Raglan sleeve t-shirt with embroidered e-textile electrode, 3. Set-in sleeve t-shirt with embroidered e-textile electrode in a random order. EMG measurements were retrieved using a wireless Delsys snap EMG sensor which was connected to the embroidered electrodes on the sleeves for t-shirt conditions and to the conventional Ag/AgCl hydrogel electrode for the bare skin condition via metal snaps. For each condition, participants engaged in a 2.5 kg dumbbell biceps curl movement which they repeated for 10 times synchronizing their bicep curl movements to a standardized metronome beat/second rhythm. In each condition, EMG data was retrieved in three repetitions of the same movement protocol to ensure a repeated measure experiment design.

In addition to the EMG measurements, electrode skin impedance data under three conditions for each participant were measured using a desktop LCR meter device (Instek LCR 6100) while the participants were in a still arm position. The measurements were performed by using a standard two-electrode configuration [22] and AC sinusoidal signal at the 0.1 to 1000 kHz frequency range. Similarly, three repetitions of electrode skin impedance per participant were employed to ensure a repeated measure experiment design. In addition, resistance and SNRdB were measured in the embroidered electrodes as well as conventional EMG electrodes.

### 2.5. Data Analysis

Collected EMG signals under three different conditions were analyzed using MATLAB according to the EMG signal forms, frequencies and SNRdB values to evaluate the effect of sleeve pattern on embroidered EMG electrode performance in comparison to conventional hydrogel electrodes applied to the bare skin.

SNRdB was calculated using the Equation (1). 8 contractions among 10 measurements of each participant, excluding the first and last trials, were used to calculate the signal Root Mean Square (RMS). RMS of the noise was calculated from the 10 s of silent muscle activity recording before the beginning of the biceps curl movement.

(1) $$\mathrm{SNR}=20*\log10\left(\frac{\mathrm{RMS}\;\left(\mathrm{Signal}\right)}{\mathrm{RMS}\;\left(\mathrm{Noise}\right)}\right)$$

Power spectral density (PSD), a tool for analysing EMG data in frequency domain characteristics, was used to compare the EMG signal further using MatLab. PSD was calculated by squaring the absolute value of Fourier Transform of EMG signal divided by the signal length. Dominant frequency, the frequency of the maximum amplitude of PSD were calculated for each trial of each participant under three conditions. One-way ANOVA and LSD post-hoc analysis were used to analyze all the data where *p* < 0.05 corresponded to significant difference between conditions.

Electrode skin impedance results for each participant under three conditions were calculated as mean ± standard deviation (SD). Since the data displayed normal distribution, both electrode–skin impedance and SNRdb measurements for embroidered electrodes on the t-shirts were analyzed using the Student’s *t*-test in comparison to conventional hydrogel Ag/AgCl electrodes. In addition, one-way ANOVA and LSD post hoc analysis were used to analyze all the data where *p* < 0.05 corresponded to significant difference between conditions.

## 3. Results and Discussion

### 3.1. Material Characterization

The wale density of the knit fabric used to prototype the t-shirts was measured as 21 wale/cm while the course density was 41.8 course/cm. As displayed in Table 2, the thickness of the fabric was 51.6 μm, the mass per unit area was 182.3 (g/m^2^) and bursting strength was 12.34 kPa. The knit fabric displayed 102.8% elasticity in wale direction and 138.3% in course direction. The residual elasticity rate measurement revealed rates of 10% in wale and 14% in course directions. Residual elasticity rate refers to recovery of the fabric or the bounce back to the original size after it has been stretched. Elasticity percentage and residual elasticity rate in course direction were used to calculate the negative ease during pattern drafting of the t-shirts with set-in and raglan sleeve variations. Negative ease refers to reducing the garment pattern dimensions so that the garment measurements are smaller than the body measurements but the elasticity of the knit fabric provides the ease and room for movement while creating a body tight fit.

Madeira HC12 conductive embroidery thread used to embroider EMG electrodes on t-shirt prototypes had 610 ± 15 dtex value and was a z-twist. The mean electrical resistivity of the thread was measured as 6.0 ± 0.0 Ω per 10 cm length. Table 3 represents the electrical characterization results of the embroidered EMG electrodes with the Madeira HC12 thread in comparison to the conventional Ag/AgCl hydrogel electrodes. Results showed significant difference between the embroidered EMG electrode and the conventional electrode where electrical resistance was lower and SNRdB was higher for the e-textile electrode.

### 3.2. EMG Measurements

**Figure 4:**
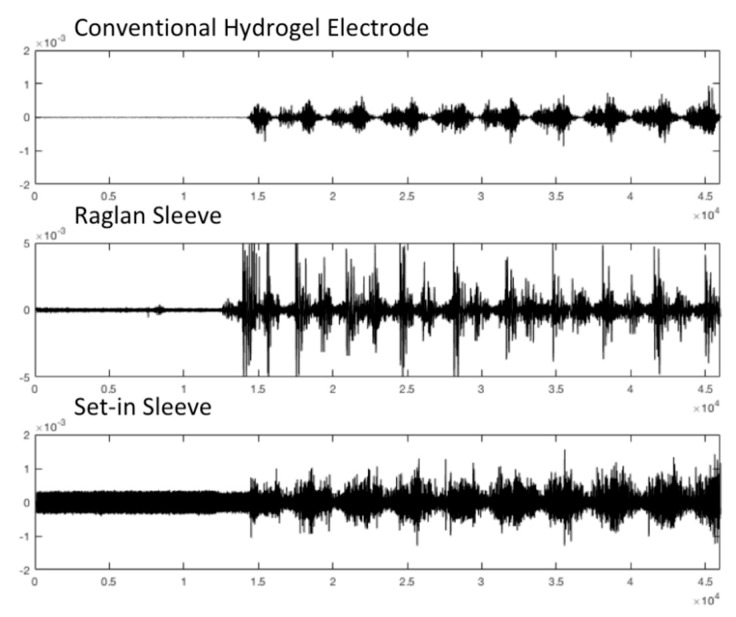
Raw EMG signals retrieved from custom fitted Participant 4 in conventional electrode and e-textile electrode in raglan and set-in sleeve conditions.

Table 4 displays the results of SNRdB and PSD analysis of the EMG signals measured while participant 1, 2 and 4 with custom fitted t-shirts engaged in the experimental protocol in bare skin with conventional hydrogel electrode, Raglan sleeve t-shirt with e-textile electrode and Set-in sleeve t-shirt with e-textile electrode conditions. Groups were tested by one-way ANOVA and post hoc LSD as displayed in Table 5 where significant difference among the groups is represented with “*” (*p* < 0.05) and results are given as Mean ± SD. Custom fit conditions were used in analyzing these results to achieve a clear comparison between hydrogel and e-textile electrodes and the two set-in and raglan sleeve garment pattern conditions. Figure 4 represents the raw EMG signal for participant 4 in different conditions as an example.

The EMG measurements retrieved with the raglan sleeve t-shirt showed similar SNRdB to the conventional hydrogel electrode. On the other hand, the set-in sleeve t-shirt with e-textile electrode had statistically significantly lower SNRdb compared to the other two conditions. The overall results for the SNDdb measurements was consistent with the literature [23]. When we evaluate the results from the perspective of garment fit and pattern, an area that has not been studied in literature, raglan sleeve pattern cut has superior signal quality compared to the set-in sleeve pattern cut in terms of EMG signal performance. Based on these findings, it is possible to discuss that the raglan sleeve pattern t-shirt, which does not have a seam directly on the shoulder joint, gets less affected from the arm movement and therefore offers continuous contact between the e-textile electrode surface and the skin. On the other hand, the seam on the shoulder joint for the set-in sleeve t-shirt does not stretch and causes temporary loss of contact between the e-textile electrode surface and the skin during movement.

**Figure 5:**
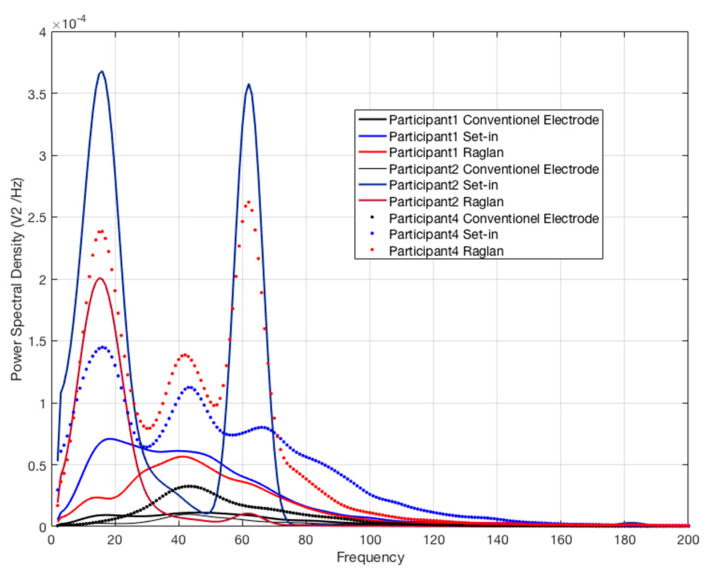
PSD distribution of EMG signals of participants with custom fit set-in sleeve, raglan sleeve and conventional electrode conditions.

Table 5 and Figure 5 display the PSD values which represent the dominant signal frequency for custom fitted Participant 1, 2 and 4. The conventional electrode shows the highest maximum frequency in PSD followed by e-textile electrodes in set-in sleeve and then the raglan sleeve condition. Frequency or spectral domain features are mostly used to study fatigue of the muscle. PSD becomes a major analysis in frequency domain and is a measure of the power that gives contribution of each frequency to the EMG signal [24,25]. In addition, Comert et al. reported that the maximum frequency (dominant frequency) of EMG signal is a parameter related to the electrode impedance [12]. It is expected to have the EMG signal frequency within the range of 0.1 to 500 Hz [26]. The maximum frequency in PSD calculated for the EMG signals retrieved in different t-shirt conditions revealed that raglan sleeve with e-textile electrodes had statistically significant lower dominant frequency compared to EMG recorded by the conventional electrodes. This calculation includes the first peak. However, e-textile electrodes recorded more than one dominant frequency in the signal with higher amplitude. In addition, the conventional electrodes almost had equally distributed frequencies in the signal, while the e-textile electrodes represented sharp pattern which resulted in narrow frequency distribution with high amplitude as seen in Figure 5. These results suggest that e-textile electrodes might detect muscle fatigue more sensitively than conventional electrodes based on PSD analysis results. Further research is needed to elucidate this finding.

**Figure 6:**
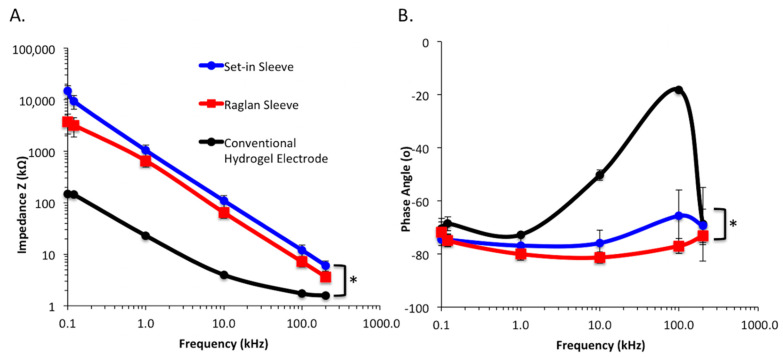
(**A**) The mean electrode–skin impedance spectrum and (**B**) the phase angle measured in different t-shirt conditions. * *p* < 0.05.

The electrode–skin impedance spectrum as displayed in Figure 6 and Table 6 was statistically significantly higher for set-in sleeve t-shirt with e-textile electrodes in comparison to the conventional hydrogel electrode on bare skin. There was also a statistically significant difference between raglan and set-in sleeve t-shirts for the electrode–skin impedance. Consistency in impedance is critical for the reliability of EMG measurements. As seen in Figure 6, even though the conventional electrode displayed a lower electrode–skin impedance at any frequency, e-textile electrodes in set-in and raglan sleeve conditions were also in the range of applicable limit for bio-electrical measurements [27]. As supported by the literature, while a low level of electrode–skin impedance is preferred, mostly the e-textile electrodes have higher impedance values compared to Ag/AgCl electrodes [27,28,29].

**Figure 7:**
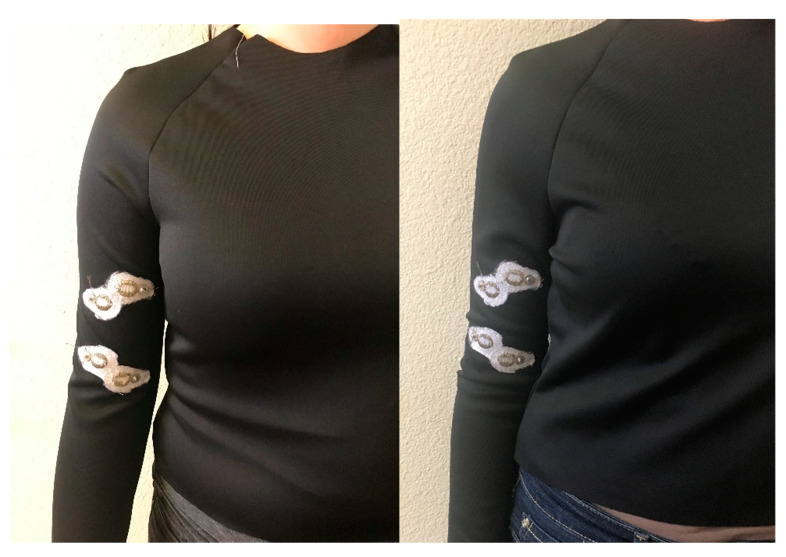
Visual display of the fit comparison for Raglan Sleeve t-shirt between Participant 2 (custom fit) and Participant 5 (slightly loose) who have same clothing size but different body forms.

In addition, the phase angle of the impedance was negative for all conditions. The average phase angle value for the conventional electrode was higher than the e- textile electrode conditions (Figure 7) Set-in and raglan sleeve conditions showed a more consistent phase angle spectrum across different frequencies compared to the conventional electrode condition. The phase angle of the conventional electrode displayed first increasing then decreasing pattern across the different frequencies. It increased from −80° at 0.1 Hz to 20° at 100 Hz. When e-textile electrodes were compared, set-in sleeve condition showed slightly high impedance in all frequencies while the raglan sleeve condition displayed lower electrode–skin impedance and more stable phase angle.

**Figure 8:**
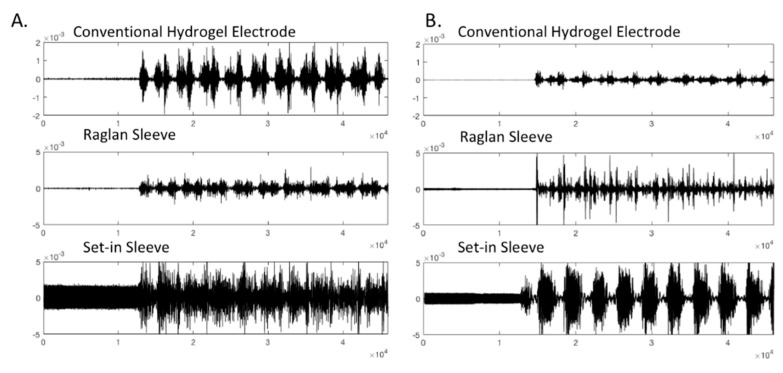
Raw EMG signals for (**A**) Participant 5 (slightly loose condition, clothing size S) (**B**) Participant 2 (custom fit condition, clothing size S) in different conditions.

In addition to analyzing the effect of different sleeve pattern cuts of set-in and raglan sleeve on EMG signal performance, the fit of t-shirts on different body types where the fit for each t-shirt pattern was either slightly snug or loose was studied (Figure 8). Table 7 shows the SNRdb measurements for raglan and set-in sleeve t-shirts where Participant 2 wears the custom fitted t-shirt and the other Participant 5 wears the same t-shirt that fits slightly loosely in comparison within the same clothing size range. The results indicated that the SNRdb value for the EMG signal is lower in the condition where the t-shirts fit loosely on the body. It is possible to discuss that tight fit of clothing on the body which does not limit joint movements leads to higher quality EMG measurements for embroidered textile-based electrodes.

**Figure 9:**
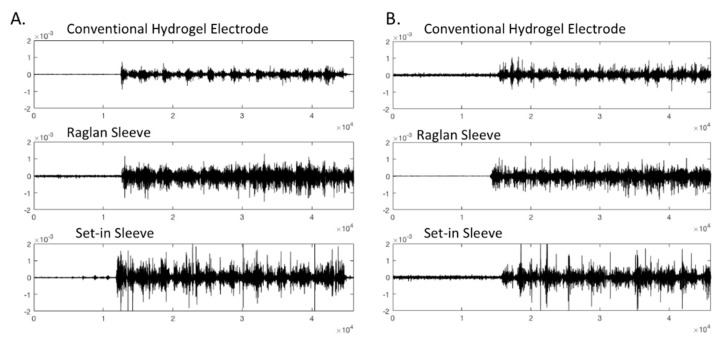
Raw EMG signal for (**A**) Participant 3 (slightly snug condition, clothing size M) (**B**) Participant 1 (custom fit condition, clothing size M) in different conditions.

Table 8 shows the SNRdb measurements and Figure 9 shows the raw EMG signal for raglan and set-in sleeve t-shirts where Participant 1 wears the custom fitted t-shirts and Participant 3 wears the same t-shirt, which fit tighter in comparison. According to the measurements, SNRdb value of the set-in sleeve condition where it fits slightly snug compared to the custom-fitted condition displayed higher results and therefore better EMG signal quality. On the other hand, for the raglan sleeve t-shirt the custom fitted condition yielded slightly higher SNRdb results in comparison to the tight-fitting condition. In this case, it is possible to argue that if the tight fit causes limitations on the movement of the joints or the t-shirt cannot freely move and stretch with the body due its tight fit, this can have negative impact on the retrieved EMG signals.

In addition, comparing raglan and set-in sleeve t-shirts from the perspective of fit and its effects on EMG performance, the data showed that set-in sleeve pattern t-shirt performed better with higher SNRdb values in snug fit condition and the SNRdb value dropped drastically in the loose condition. On the other hand, the raglan sleeve pattern t-shirt did not display any drastic changes in EMG signal quality in loose and snug conditions but performed best in the custom-fit condition. Based on this comparison, it is possible to discuss that raglan-sleeve pattern could be less affected by slight fit variations within the same size range and set-in sleeve pattern should be developed with extra negative-ease for better performance.

## 4. Conclusions

This study investigated the effect clothing pattern and fit in EMG applications using e-textile electrodes integrated onto the sleeves of custom drafted t-shirts with set-in and raglan sleeves. The raglan sleeve pattern, widely used in athletic wear to provide extra ease for the movement of shoulder joint, showed superior performance and therefore indicated the pattern of a garment could have significant effect on EMG signal quality in the design of smart clothing. The findings of the study can be specifically summarized in four points.

1. Embroidered e-textile EMG electrode performance is directly related to clothing pattern and cut. The raglan sleeve pattern, which does not have a seam directly on the shoulder joint, shows significantly higher signal-to-noise ratio and gets less affected from the arm movement.
2. The frequency distribution of the PSD analysis indicated that the e-textile electrodes might detect muscle fatigue more sensitively; however, this finding requires further research.
3. The electrode–skin impedance spectrum analysis of e-textile electrodes in raglan sleeve condition shows a more consistent impedance spectrum across different frequencies and therefore relates to considerably higher signal-to-noise ratio of the measured EMG signal.
4. Embroidered e-textile EMG electrode performance is directly related to how clothing fits on the body. Analysis done in comparison to custom-fit set-in and raglan sleeve pattern conditions revealed that slightly snug or slightly loose fit conditions within the same clothing size range results in decline in the EMG signal quality for the raglan-sleeve pattern style. On the other hand, the set-in sleeve pattern style performs better when the fit is more snig and the EMG signal quality significantly declines in loose conditions.

In addition to these findings, there are some limitations of the study that can pave way into future research. Increasing the number of participants can provide a comparison across genders. In this study, even though the data does not show any tendency to be different across genders according to the SNRdb data, it was not possible to draw a generalizable conclusion. In addition, investigation of fabric displacement during movement as well as applied pressure by clothing across different pattern and fit conditions can provide a more in-depth understanding on the role of clothing pattern and cut in smart clothing development for EMG applications.

This pioneering preliminary study proves the direct relationship between e-textile electrode performance and clothing pattern and cut and therefore the clothing fit. E-textile EMG electrodes have superior signal quality when t-shirt patterns and sizing are carefully designed, consider the movements of the body and alignment of the seamlines, as well as the elasticity and recovery of the fabric to create the optimum fit in dynamic conditions.

## Figures and Tables

**Table 1:** Demographic information, clothing size and BMI measurements of participants.

Participant	Gender	Age	BMI	Clothing Size	Prototype Fit Condition	Armhole Girth (cm)	Bicep Girth (cm)	Shoulder Width (cm)	Chest/Bust Girth (cm)
1	Male	39	25.2	M	Custom Fit	53.84	31.95	51.38	99.08
2	Female	26	23.4	S	Custom Fit	41.19	11.64	37.84	92.07
3	Male	43	25.8	M	Snug compared to Participant 1	54.62	32.58	51.76	101.08
4	Female	22	19.8	S	Custom Fit	39.49	10.49	36.65	85.09
5	Female	38	21.1	S	Loose compared to Participant 2	40.79	10.72	37.16	83.21
**Participant**	**Gender**	**Age**	**BMI**	**Clothing Size**	**Prototype** **Fit Condition**	**Armhole Girth (cm)**	**Bicep Girth (cm)**	**Shoulder Width (cm)**	**Chest/Bust** **Girth (cm)**
1	Male	39	25.2	M	Custom Fit	53.84		51.38	99.08
2	Female	26	23.4	S	Custom Fit	41.19		37.84	92.07
3	Male	43	25.8	M	Snug compared to Participant 1	54.62		51.76	101.08
4	Female	22	19.8	S	Custom Fit	39.49		36.65	85.09
5	Female	38	21.1	S	Loose compared to Participant 2	40.79		37.16	83.21

**Table 2:** Mechanical characteristics of the knit fabric used for t-shirt prototypes.

Thickness (μm)	Weight (g/m^2^)	Elasticity (%)		Residual Elasticity (%)		Bursting Strength (kPa)
		Wale	Course	Wale	Course	
51.6	182.3	120.8	138.3	10	14	12.34

**Table 3:** Electrical characterization of embroidered and conventional EMG electrodes. Results are given as Mean ± SD.

	Mean R (Ω)	SNR (dB)
Embroidered Electrode	0.087 ± 0.005	70.83
Conventional Electrode	649.33 ± 145.14	60.63

**Table 4:** Analysis of EMG signals’ SNRdb and maximum frequency in PSD. Results are given as Mean ± SD.

	SNR (dB)	Maximum Frequency in PSD (Hz)
Conventional Hydrogel Electrode	21.8 ± 8.9	45 ± 4
Raglan Sleeve t-shirt, Embroidered Electrode	22.5 ± 8.1	25 ± 7 *
Set-in Sleeve t-shirt, Embroidered Electrode	14.0 ± 7.6 *	37 ± 2

* *p* < 0.05.

**Table 5:** One-way ANOVA and post-hoc LSD result of SNRdB values.

		Mean Difference	Std. Error	Significance	95% Lower Bound	95% Upper Bound
Conventional Electrode	Raglan Sleeve Embroidered Electrode	−0.675	3.669	0.855	−8.17	6.82
	Set-in Sleeve Embroidered Electrode	7.742 *	3.533	0.037	0.51	14.96
Raglan Sleeve Embroidered Electrode	Conventional Electrode	0.675	3.669	0.855	−6.82	8.17
	Set-in Sleeve Embroidered Electrode	8.418 *	4.072	0.048	0.089	16.74
Set-in Sleeve Embroidered Electrode	Conventional Electrode	−7.742 *	3.533	0.037	−14.96	−0.51
	Raglan Sleeve Embroidered Electrode	−8.418 *	4.072	0.048	−16.74	−0.08

* *p* < 0.05.

**Table 6:** One-way ANOVA and post-hoc LSD result of electrode–skin impedance values.

		Mean Difference	Std. Error	Significance	95% Lower Bound	95% Upper Bound
Conventional Electrode	Raglan Sleeve Embroidered Electrode	−1212.79	922.079	0.201	−3115.87	690.28
	Set-in Sleeve Embroidered Electrode	−4134.92 *	922.079	0.000	−6038.00	−2231.84
Raglan Sleeve Embroidered Electrode	Conventional Electrode	1212.79	922.079	0.201	−690.28	3115.87
	Set-in Sleeve Embroidered Electrode	−2922.13 *	922.079	0.004	−4825.20	−1019.05
Set-in Sleeve Embroidered Electrode	Conventional Electrode	4134.92 *	922.079	0.000	2231.84	6038.00
	Raglan Sleeve Embroidered Electrode	2922.12 *	922.079	0.004	1019.05	4825.20

* *p* < 0.05.

**Table 7:** SNRdb values for raglan and set-in sleeve t-shirts for Participant 2 and Participant 5 displaying custom-fit and slightly loose condition.

	Raglan Sleeve T-Shirt SNR (dB)	Set-in Sleeve T-Shirt SNR (dB)
Participant 2 (custom fit condition, clothing size S)	22.2 ± 8.8	16.4 ± 7.7
Participant 5 (slightly loose condition, clothing size S)	17.3 ± 5.5	5.1 ± 2.0

**Table 8:** SNRdb values for raglan and set-in sleeve t-shirts for Participant 1 and Participant 3 displaying custom-fit and slightly snug condition.

	Raglan Sleeve T-Shirt SNR (dB)	Set-in Sleeve T-Shirt SNR (dB)
Participant 1 (custom fit condition, clothing size M)	26.2 ± 2.4	17 ± 1.9
Participant 3 (slightly snug condition, clothing size M)	22.1 ± 3.4	22.1 ± 1.7
